# Your critical care patient may have HLH (hemophagocytic lymphohistiocytosis)

**DOI:** 10.1186/s13054-016-1369-3

**Published:** 2016-07-06

**Authors:** Rafal Machowicz, Gritta Janka, Wieslaw Wiktor-Jedrzejczak

**Affiliations:** Department of Hematology, Oncology and Internal Diseases, Medical University of Warsaw, ul. Banacha 1a, 02-097 Warsaw, Poland; Department of Paediatric Haematology and Oncology, University Medical Centre Hamburg Eppendorf, Martinistrasse 52, 20246 Hamburg, Germany

Among various actions taken to improve the prognosis of critical care patients, an important step is including hemophagocytic lymphohistiocytosis (HLH; hemophagocytic syndrome) in the differential diagnosis.

HLH is an uncontrolled, self-propelling hyperinflammation. It can be initiated in a predisposed individual by various triggering factors: infection (especially viral, notably Epstein-Barr virus (EBV)), malignancy (frequently by T-cell lymphoma), or autoimmune disease. The triggering factor unleashes a positive feedback loop with extremely high concentrations of pro-inflammatory cytokines (“cytokine storm”) which leads to multiorgan failure (MOF) and death. Only timely proper diagnosis and treatment can reverse this dismal outcome.

The cause of this rare, pathologic hyperinflammation is an immune dysfunction, which in familial cases was found to result from an inherited lack of cytotoxic activity of T cytotoxic and natural killer lymphocytes, which retain the ability to release cytokines. A similar, but not fully understood, mechanism is expected in the acquired cases. Mutations in cytotoxic granule pathway genes (e.g., *Perforin*, *UNC13D* or *STX11*) are the cause in familial HLH [1]. HLH was at first perceived as affecting only infants with mutations in these specific genes, but later the syndrome was also found in adolescents and adults, so the term “secondary HLH” was coined. This approach was abandoned because (regardless of age and mutation status) all HLH episodes are thought to be secondary to a triggering factor (which cannot always be identified). Nowadays, with a growing number of adult patients of whom a considerable proportion harbor a proven mutation in HLH-related genes (25 % [2], 14 % [3]), the syndrome is considered as a continuum from neonates with complete loss of cytotoxic function, through adolescents to elderly adults with mutations only partially affecting the cytotoxic activity or patients without any characteristic mutation but with symptomatic HLH.

In most patients, in the absence of genetic traits, diagnosis of HLH is based on fulfillment of five out of eight HLH 2004 [1] criteria: 1) persistent fever; 2) splenomegaly; 3) bicytopenia (hemoglobin <9 g/dl, neutrophils <1.0 × 10^9^/l, platelets <100 × 10^9^/l); 4) hypofibrinogenemia (<150 mg/dl) and/ or hypertriglyceridemia (>265 mg/dl); 5) hyperferritinemia (>500 ng/ml); 6) hemophagocytosis; 7) low natural killer cell activity; and 8) high concentration of sCD25 (soluble receptor for interleukin 2).

Hemophagocytosis occurs when activated macrophages phagocytose other blood or bone marrow cells. Despite the name of the syndrome, hemophagocytosis is neither necessary nor sufficient to diagnose HLH—it is only one of the criteria. It is not an essential part of the HLH pathomechanism, but rather develops in a more advanced stage of the process [4]. Therefore, it can be absent in patients with HLH, and it is also not pathognomonic for this syndrome. Hemophagocytosis is a frequent finding, especially in intensive care—it was observed in 64 % of the ICU patients with sepsis and thrombocytopenia [5] (32/50) and in 65 % deceased ICU patients [6] (69/107). Diagnosis of HLH is possible without bone marrow aspirate/biopsy; however, this procedure should be performed to search for underlying malignancy. Neoplasms are among the most important HLH triggers in adults and are associated with a poor prognosis [7].

The most important parameter, besides cytopenias, characteristic for HLH is hyperferritinemia. The specific (especially for ICU patients) values are much higher than the HLH 2004 criterion and exceed 2000–3000 ng/ml, reaching high specificity (but low sensitivity) above 10,000 ng/ml. All patients with such high ferritin values should have other HLH criteria assessed. Indications for ferritin testing are cytopenias and/or splenomegaly in a febrile patient whose clinical status and laboratory values are worsening despite standard treatment with anti-infectious therapy.

Although treatment of the trigger is important (if it is possible at all), this is usually not sufficient to suppress the high cytokine levels. Treatment modalities include immunosuppressive/immunomodulatory agents such as steroids, intravenous immunoglobulins, cyclosporine A, removal of the cytokines by plasmapheresis, and, in severe cases, etoposide [8]. A delay in etoposide treatment was proven to have a negative impact on prognosis in EBV-associated HLH [9]. Without immune suppression, and despite all possible efforts of intensive care, HLH is often fatal.

Raising the awareness of HLH—a life-threatening, heavily underdiagnosed syndrome—is of paramount importance, especially since the tests for preliminary diagnosis (ferritin concentration) and treatment (etoposide, steroids, cyclosporine A) are relatively inexpensive and widely available.

## Abbreviations

EBV, Epstein-Barr virus; HLH, hemophagocytic lymphohistiocytosis; ICU, intensive care unit; MOF, multiorgan failure
